# On the Stationarity Time of a Vehicle-to-Infrastructure Massive Radio Channel in a Line-of-Sight Suburban Environment

**DOI:** 10.3390/s22218420

**Published:** 2022-11-02

**Authors:** Nor El Islam Dahmouni, Pierre Laly, Marwan Yusuf, Gauthier Delbarre, Martine Liénard, Eric P. Simon, Davy P. Gaillot

**Affiliations:** 1 Univ. Lille, CNRS, UMR 8520—IEMN, F-59000 Lille, France; 2 Department of Information Technology IMEC-WAVES, Ghent University, 9052 Gent, Belgium

**Keywords:** V2I, 5G, stationarity time, GLSF, channel correlation function, collinearity, massive MIMO

## Abstract

Massive multiple-input multiple-output (mMIMO) communication systems are a pillar technology for 5G. However, the wireless radio channel models relying on the assumption of wide-sense stationary uncorrelated scattering (WSSUS) may not always be valid for dynamic scenarios. Nonetheless, an analysis of the stationarity time that validates this hypothesis for mMIMO vehicular channels as well as a clear relationship with the scattering properties is missing in the literature. Here, time-varying single-user mMIMO radio channels were measured in a suburban environment at the 5.89 GHz vehicular band with a strong Line-of-Sight (LOS) to study the non-WSSUS and large scale characteristics of the vehicle-to-infrastructure (V2I) link. The generalized local scattering function (GLSF), computed from the sampled channels, was used to derive (1) the spatial distribution of the stationarity time using the channel correlation function (CCF) and empirical collinearity methods and (2) the root mean square delay/angular spread and coherence time/bandwidth values from the projected power delay profile (*PDP*) and Doppler power spectra (*DPS*). The results highlight the high degree of correlation between the spatial distribution of the stationarity time and the scattering properties along the measurement route.

## 1. Introduction

Recently, the fifth generation (5G) technology has been revolutionizing the development of intelligent transportation systems (ITS) for vehicle-to-everything (V2X) communication technology. Specifically, with the launch of 5G-based vehicular technologies such as ITS-G5 (based on IEEE 802.11p) and cellular vehicle-to-everything C-V2X (developed by 3rd Generation Partnership Project), the study of wireless vehicular communications has become a hot research topic [1,2,3]. To support the 5G requirements, massive multiple-input multiple-output (mMIMO) with many antennas at the transmitter side has emerged as a key technology to improve the network quality of service with increased capacity and overall user experience [4]. mMIMO 5G base stations are now typically equipped with 64 fully digital transceivers that deliver large Signal-to-Noise Ratio (SNR) values through a better beam management. This is specifically relevant when a dominant line-of-sight (LOS) exists between the transmitter and receiver [4] and if the propagation characteristics do not evolve too rapidly over time. Hence, the characterization of the time-varying wireless propagation channel is a crucial step to develop robust and reliable vehicular communication systems that serve the requirement of ITS in terms of safety and security under the umbrella of ultra-reliable low latency communications (URLLC) [3]. In particular, the main communicating link between the vehicle and road-side units connected to the core networks (V2I) is of interest.

A key feature for designing and analyzing wireless propagation channels is the assumption of wide-sense stationary and uncorrelated scattering (WSSUS). This implies that the second-order channel statistics, but not the channel realizations, are independent of time and center frequency [5]. However that hypothesis, in particular the WSS one [5,6], is not always applicable for V2X scenarios since the correlation function becomes time- and frequency-dependent. This effect is due to the high mobility of the receiver (Rx) with respect to the transmitter (Tx) and the presence of dynamic scatterers. In fact, the WSS violation occurs due to the time-varying nature of the multipath components (MPC) statistics [7,8]. The US assumption is violated if channels exhibit correlated scattering due to several MPC that are close in the delay-Doppler domain resulting from the same physical scatterer, or delay/Doppler leakage due to bandwidth/time limitations at Tx or Rx. From a functional perspective, the analysis of non-stationary statistics could help with optimizing adaptive transmission schemes and beamforming techniques [9], as well as improving the overall system performance [6,8]. Nonetheless, only MIMO radio channels have been investigated so far [9].

### 1.1. Related Work

It follows that the non-stationary fading process of vehicular channels can be characterized by assuming local stationarity for a finite region in time and frequency. A definition of the stationarity time and stationarity bandwidth is proposed in [10,11] where a comprehensive framework was developed. Basically, non-WSSUS channels can be studied by extending the WSSUS scattering function C(ν,τ) that depends on Doppler ν and delay τ, to the the time- and frequency-dependent generalized local scattering function (GLSF) $\hat{C}\left(t,f,\nu ,\tau \right)$. The stationarity region defined by its stationarity time and frequency bounds $T_{s}$ and $F_{s}$, respectively, can be subsequently derived from the GLSF using different approaches. The collinearity method was used by [5,12] to study the non-WSSUS vehicular channel characteristics and validity in various scenarios at 5.2 GHz. In [9,13,14], the authors reported on the analysis of non-WSSUS MIMO vehicular communications using the correlation matrix distance as an empirical approach developed in [9]. The correlation matrix distance allows for tracking changes in the full spatial structure of non-stationary MIMO channels by measuring the similarity between two correlation matrices (two covariance matrices) at two different times. The stationarity of 5×5 MIMO vehicular channels has also been studied in [15,16] using either the collinearity between power delay profiles (PDPs) and GLSFs, respectively.

In addition, a MIMO radio channel sounder operating at 1.35 GHz with an 80 MHz bandwidth was used in our previous works treating V2I radio channels. A deep analysis of the stationarity time was reported in a suburban [6] and tunnel environment [17]. We also proposed a framework for long-term vehicular channel simulation based on the vector time-frequency autoregressive model for a sparse parametric description of non-stationary multivariate random processes [18]. This approach was found as an efficient alternative for non-stationary channel simulation that is measurement-based and computationally inexpensive.

From the literature point of view, it can be safely concluded that a study of mMIMO vehicular radio channels emphasizing on the channel stationarity characteristics is currently missing.

### 1.2. Contribution of This Work

In this paper, it is proposed to fill this gap by using our most recent radio channel system that has been upgraded and validated to assess the time-varying characteristics of 64 × 1 mMIMO radio channels around the ITS frequency band [19]. The originality and main contributions are the following:

- The statistical spatial behavior of the stationarity time $T_{s}$ across the 64 Tx antennas of the massive MIMO system is provided along the measurement route.
- It is shown that the spatial distribution of $T_{s}$ across the massive array is strongly correlated along the array vertical dimension justifying mMIMO linear arrays.
- The analysis reveals that this behavior is highly correlated with the large scale channel parameters properties along the measurement route.

The paper is organized as follows: the methodology presented in Section 2 includes the setup and measurement scenarios as well as a description of the GLSF estimation and preprocessing. Before concluding, the stationarity time and radio channel characteristics are discussed in Section 3.

## 2. Methodology

**Figure 1:**
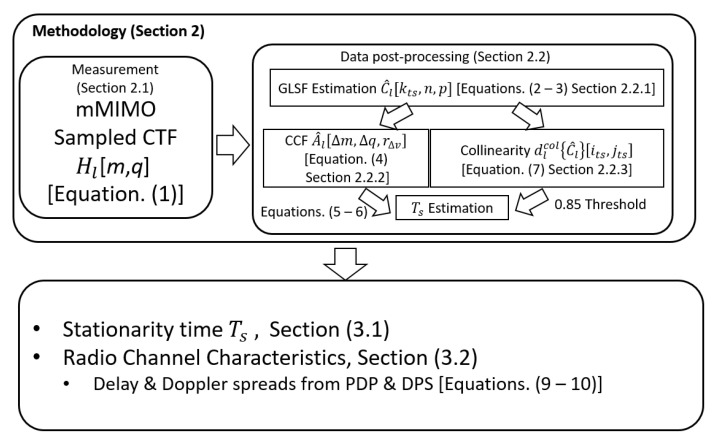
Work methodology.

Figure 1 illustrates the methodology followed in this work to obtain the presented results. First, the sampled channel transfer function (CTF) is measured for the investigated scenario using a dedicated mMIMO radio channel sounder (Section 2.1). Then, the GLSF, computed from the CTF using orthogonal multi-window tapers, is used to derive the stationarity time using the collinearity and CCF methods as detailed in Section 2.2. In addition, the *PDP* and *DPS* were calculated by projecting the GLSF in the delay and Doppler dimension, respectively, to obtain estimates of the large scale parameters: delay/Doppler spreads, coherence time and bandwidth.

### 2.1. Measurement Setup and Scenario

**Figure 2:**
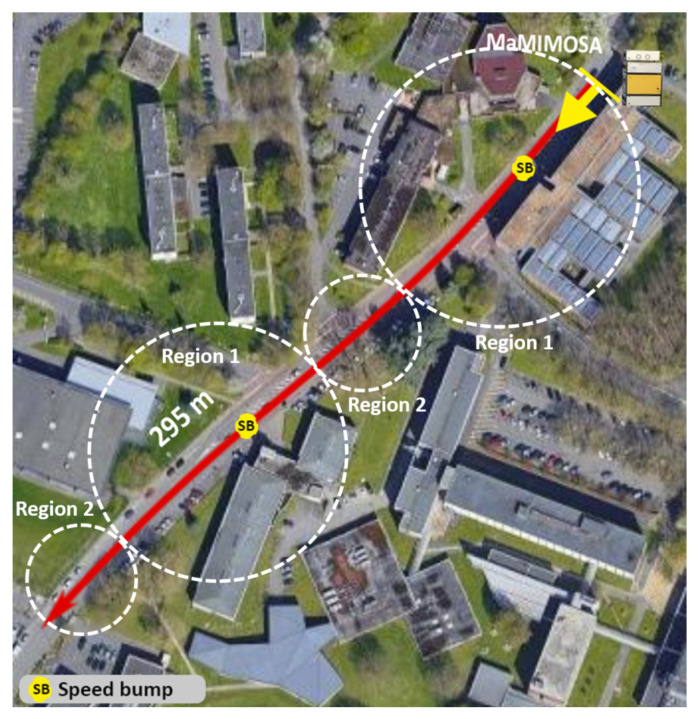
Top view of the V2I measurement campaign scenario at the University of Lille campus. The dashed circles indicate the two identified regions with distinct propagation characteristics.

**Figure 3:**
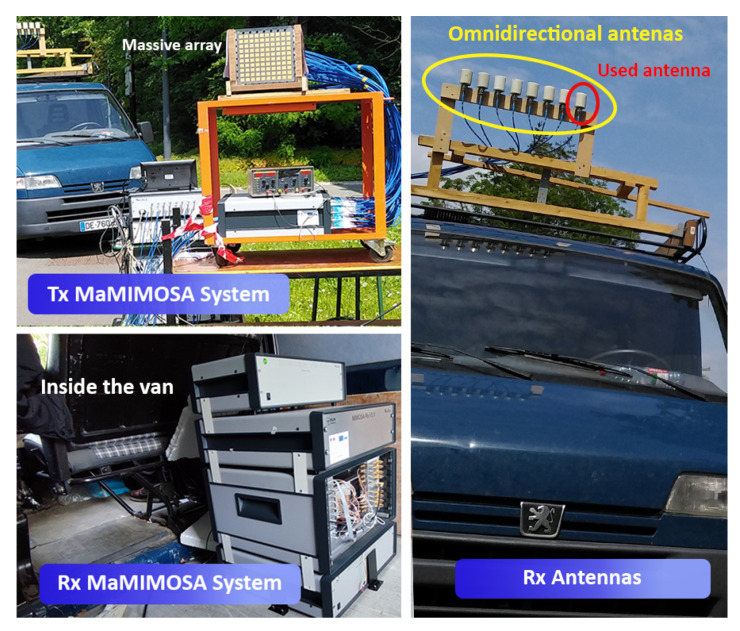
Montage of the Tx and Rx MaMIMOSA system showing the massive Tx array and Rx omnidirectional antenna.

A radio channel measurement campaign was carried out on the campus of Lille University (France) and is presented in Figure 2. This suburban environment is comprised of 3–4 floors buildings spaced 5 to 10 m apart, with dense vegetation with trees up to 30 m high. There is relatively low traffic on the road, with a maximum speed of 50 km/h, but many cars were parked along the boulevard. The Tx is a massive square array of $M_{Tx}=64$ antennas and was installed as a road-side unit (RSU) on the sidewalk at two meters high above the ground. An omnidirectional Rx antenna was placed on the roof of a van at 3 m high, as illustrated in Figure 3. The van was moving away from Tx at a constant 40 km/h speed over a total distance of 295 m, such that an LOS was always present, as shown in Figure 2. The measurement was performed by the real-time radio channel sounder MaMIMOSA with an 80 MHz bandwidth at 5.89 GHz [19,20] corresponding to the frequency band offered by ITS derived from the frequency band 5.9 GHz ITS-G5 and C-V2X technologies.

**Figure 4:**
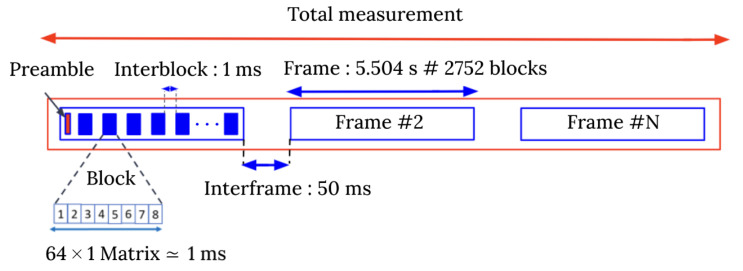
MaMIMOSA frame structure configuration.

The MaMIMOSA streaming mode was selected to measure the time-varying CTF H(t,f) of the massive radio channel [19] between the massive transmitter and single-antenna receiver (i.e., downlink). It is associated with the frame structure illustrated in Figure 4. Each frame consists of one 51.2 µs preamble followed by $M_{t}=2752$ blocks of eight orthogonal frequency-division multiplexing (OFDM) symbols. The preamble provides the time synchronization of the frame required to decode the OFDM symbols. Each symbol corresponds to a row of the 8 × 8 massive array and has a duration of 121.92 µs, including the 40 µs cyclic prefix. The automatic gain control (ACG) is performed with the cyclic prefix for each symbol and is used to correct the stored measured complex MIMO matrix. The total number of transmitted OFDM subcarriers per symbol is 8192, which can be uniformly distributed on each antenna element using interleaved OFDM. The frequency space between subcarriers is 12.21 kHz, in line with the basic 4G and 5G numerology. For each Tx-Rx link, $M_{f}=818$ frequency samples spaced by eight subcarriers were selected across the 80 MHz bandwidth. The whole array is spanned by sequentially switching between the eight rows. Hence, a 64×1 mMIMO matrix called block is measured in ∼1 ms, and the frame duration is 5.504 s. The maximum Doppler span is ±250 Hz (∼46 km/h maximum speed) with 0.181 Hz Doppler resolution. The time duration between consecutive blocks and frames was manually set to 1 ms and 50 ms, respectively. Five frames were measured during the driving test giving a measurement time total of 27.18 s and a total of 13,760 blocks (i.e., 880640 CTF). The total number of frames that can be measured by the system solely depends on the available hard drive space. For each Tx-Rx link *l*, the sampled CTF can be represented as follows:

(1) $$H_{l}\left[m,q\right]=H_{l}\left(t_{s}m,f_{s}q\right),$$

where $t_{s}=1.9753$ ms is the block sampling rate time and $f_{s}=97.79$ KHz is the frequency resolution with $m\in \{0,...,M_{t}-1\}$, $q\in \{0,...,M_{f}-1\}$ and l∈{1,...,64}. Table 1 summarizes the MaMIMOSA parameters configuration.

### 2.2. Evaluation of the Stationarity Time

For similar scenarios, it was reported in [5] that the frequency stationarity region was assumed to be the minimum value of $F_{s}=150$ MHz. Here, since the measurement bandwidth is 80 MHz, it is considered that the radio channel is stationary in frequency. Hence, only the stationarity time is investigated in this work. It is noteworthy that this is intrinsically true for all ITS technologies at 5.9 GHz since channels typically occupy less than 20 MHz. Typically, the stationarity time $T_{s}$ can be derived using two approaches that both require the computation of the GLSF. The first approach relies on CCF, which is the three-dimensional Fourier transform of the GLSF. The stationary time region is estimated from the CCF using the method of discrete Doppler correlation as explained in [10,11]. The Doppler CCF quantifies the Doppler lag interval in which a significant correlation exists. This interval corresponds to the stationary time in the dual time-frequency domain. For the second approach, $T_{s}$ is empirically estimated using the collinearity method. The collinearity is first computed as the correlation between GLSF sequences. $T_{s}$ is then given as the region where the collinearity values cross a pre-defined threshold between 0 and 1 [12], where 1 indicates identical similarity, and 0 indicates high dissimilarity [5].

#### 2.2.1. Computation of the GLSF

In the following, the calculation of the GLSF is detailed. Based on [5,6,10,11], the fading process was estimated as locally stationary in time for a region of $M_{ts}=86$ blocks equivalent to 169.88 ms ($M_{ts}\times t_{s}$). For each Tx-Rx link *l*, I×J orthogonal multi-window tapers $G_{w}$ in time (*I*) and frequency (*J*) (w∈{0,…,IJ−1}) are chosen from discrete prolate spheroidal sequences (DPSS) to obtain multiple independent realizations [11,12]. In other words, the GLSF is computed by performing a local filtering in time and frequency with orthogonal deterministic functions.

The sampled $k_{ts}^{th}$ GLSF ${\hat{C}}_{l}\left[k_{ts},n,p\right]$ is calculated as follows:

(2) $${\hat{C}}_{l}\left[k_{ts},n,p\right]=\frac{1}{IJ}\sum_{w=0}^{IJ-1}|H_{l}^{\left(G_{w}\right)}\left[k_{ts},n,p\right]{|}^{2},$$

where $n\in \{0,...,M_{f}-1\}$ denotes the delay index and $p\in \{-M_{ts}/2,...,M_{ts}/2-1\}$ denotes the Doppler index. Hence, the local Doppler resolution is given by $\nu_{s}=1/\left(M_{ts}t_{s}\right)=5.88$ Hz. The multi-window function $H^{\left(G_{w}\right)}$ is estimated as:

(3) $$\begin{array}{c} H_{l}^{\left(G_{w}\right)}\left[k_{ts},n,p\right]=\sum_{q^{{}^{\prime }}=-M_{f}/2}^{M_{f}/2-1}\;\sum_{m^{{}^{\prime }}=-M_{ts}/2}^{M_{ts}/2-1}G_{w}\left[m^{{}^{\prime }},q^{{}^{\prime }}\right]\times \\ H_{l}\left[m^{{}^{\prime }}+\Delta_{t}k_{ts}+M_{ts}/2,q^{{}^{\prime }}+M_{f}/2\right]e^{-j2\pi (pm^{{}^{\prime }}-nq^{{}^{\prime }})}, \end{array}$$

where $m^{\prime }$ and $q^{\prime }$ represent the relative time and frequency indices, respectively. $\Delta_{t}$ denotes the time shift between consecutive locally stationary regions. The window $G_{w}$ is localized in $\left[-M_{ts}/2,M_{ts}/2-1\right]\times \left[-M_{f}/2,M_{f}/2-1\right]$. To balance the variance and bias as shown in [11], I=J=3 for both time and frequency.

#### 2.2.2. Computation of $T_{s}$ Using the Discrete CCF Method

Following the computation of the GLSF, the discrete CCF can be obtained as its three-dimensional Discrete-time Fourier Transform (DTFT) [6,10]:

(4) $${\hat{A}}_{l}\left[\Delta m,\Delta q,r_{\Delta \nu }\right]=F^{3}\left\{{\hat{C}}_{l}\left[k_{ts},n,p\right]\right\},$$

where Δm, Δq and $r_{\Delta \nu }$ represent the time lag, frequency lag, and Doppler lag indices, respectively. Subsequently, the discrete Doppler correlation $\bar{{\left(r_{\Delta \nu }\right)}_{l}}$ is deduced from the CCF Doppler first moment as follows [6,10]:

(5) $$\bar{{\left(r_{\Delta \nu }\right)}_{l}}=\frac{1}{\left|\right|{\hat{A}}_{l}{\left|\right|}_{1}}\sum_{r_{\Delta \nu }}\sum_{\Delta q}\sum_{\Delta m}\left|r_{\Delta \nu }\right|{\hat{A}}_{l}\left[\Delta m,\Delta q,r_{\Delta \nu }\right],$$

where $\left|\right|\hat{A}{\left|\right|}_{1}$ is the first norm of the CCF along the three dimensions. Finally, the stationarity time $T_{s}$ can be calculated for each Tx-Rx link *l* using the formula given in [10]:

(6) $${\left(T_{s}\right)}_{l}=\frac{1}{\bar{{\left(r_{\Delta \nu }\right)}_{l}}}.$$

#### 2.2.3. Computation of $T_{s}$ Using the Collinearity Method

For the second approach, the collinearity $d_{l}^{col}$ between GLSFs sampled at two different times $i_{ts}$ and $j_{ts}$ is calculated for each Tx-Rx link *l* as follows [5,12]:

(7) $$d_{l}^{col}\{{\hat{C}}_{l}\}\left[i_{ts},j_{ts}\right]=\frac{tr\{{\hat{C}}_{l}\left[i_{ts},n,p\right]{\hat{C}}_{l}\left[j_{ts},n,p\right]\}}{\left|\right|{\hat{C}}_{l}\left[i_{ts},n,p\right]{\left|\right|}_{F}\left|\right|{\hat{C}}_{l}\left[j_{ts},n,p\right]{\left|\right|}_{F}},$$

where tr{X} and ${\left||X|\right|}_{F}$ denote the trace and Frobenius norm of *X*, respectively. $T_{s}$ defines the region for which the collinearity values cross a pre-defined threshold selected to 0.85 in this work [5,6,12].

## 3. Results

### 3.1. Stationarity Time

**Figure 5:**
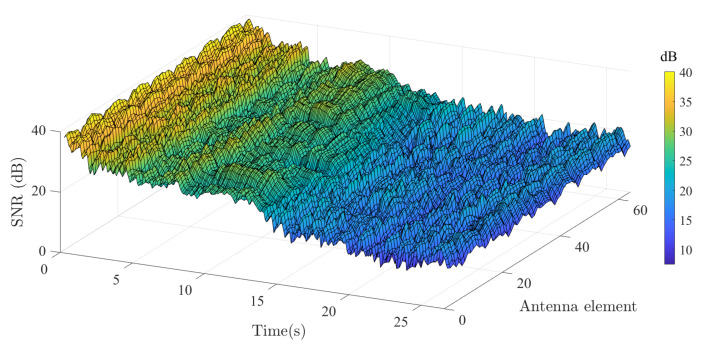
Computed SNR as a function of antenna and time index.

Here, the GLSF was derived from the measured CTF, and $T_{s}$ was computed as shown in Section 2.2 using 10 interleaved GLSF corresponding to 934.34 ms using the two methods (i.e., CCF and collinearity) described earlier. This value is deliberately larger than the local stationarity time of 567 ms reported in [6] for a similar scenario. In order to obtain faithful $T_{s}$ estimate values across the whole array, the signal-to-noise ratio (SNR) values were calculated from the GLSF. As it is shown in Figure 5, the SNR level continuously decreases since the van is moving away from Tx with values ranging from 40 to 10 dB. The SNR is found to be spatially uniform with time-averaged deviation <3 dB and median =1.70 dB. This also indicates that no significant masking effects occurred during the measurement scenario.

**Figure 6:**
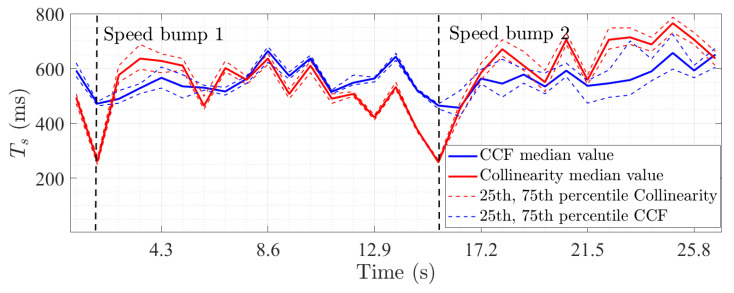
Spatial distribution of $T_{s}$ obtained from the CCF (blue) and collinearity (red) methods. The median value is shown with a solid line and the 25th/75th percentiles with a dashed line.

Figure 6 presents the 25th, 75th percentiles and median value computed from the spatial distribution of $T_{s}$ using both methods. A 0.9 threshold was used for the collinearity method. The results show an averaged median value for $T_{s}$ of 568 ms and 565 ms for the CCF and collinearity approaches, respectively, indicating that, on average, these two methods behave similarly. Nonetheless, it is observed for two specific time frames that the $T_{s}$ values obtained with the collinearity method are much lower than those computed from the CCF method. This effect is attributed to the presence of two speed bumps along the driving test shown in Figure 2. Indeed, the position of the Rx antenna was brutally modified during a short time of around 100 ms less than the CCF window. It is noteworthy that the collinearity method highlights this rapid change well in the propagation channel statistics, whereas the CCF does not. The discrepancy between the results of the two methods was discussed in [6]. Furthermore, for both methods, the $T_{s}$ values are also observed to be spatially uniform during the first second and between ∼9 and ∼13 s. This can be explained by the scattering properties of the environment, which will be discussed in more detail later.

**Figure 7:**
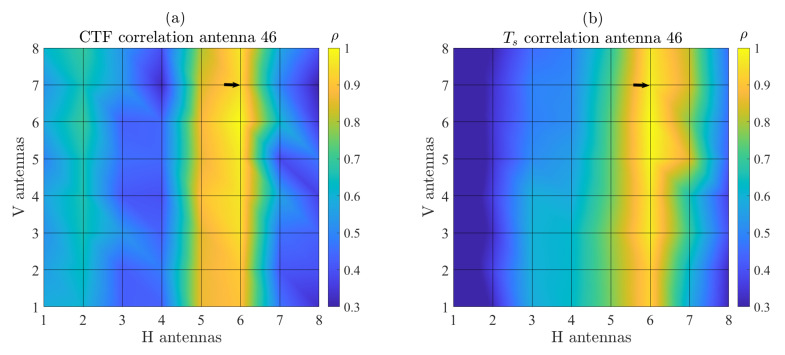
Spatial correlation computed from (**a**) the time-varying Ts values and (**b**) CTF for antenna #46 (black arrow).

**Figure 8:**
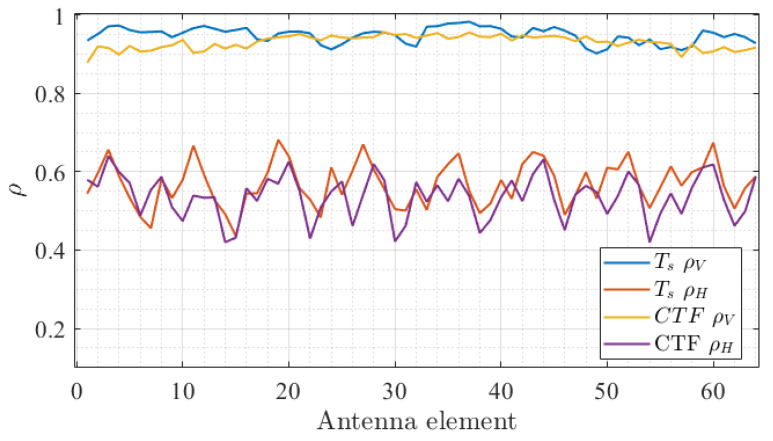
Average $\rho_{V}$ and $\rho_{H}$ computed from the time-varying $T_{s}$ values (**blue and yellow plot**) and CTF (**red and purple plot**) as a function of the antenna index.

In addition, the spatial distribution of $T_{s}$ computed using the CCF method was investigated with greater depth across the array by evaluating its correlation properties. For each antenna, the $T_{s}$ values over the total measurement time were stacked into a vector. Then, the correlation between a reference antenna vector and all other antenna vectors was computed. As an example, Figure 7a presents the results of this analysis for the arbitrary antenna #46 (indicated by the black arrow). The data show that $T_{s}$ is strongly correlated along the vertical (V) axis compared to the horizontal (H) axis with average values of $\rho_{V}=0.948$ and $\rho_{H}=0.569$, respectively. This indicates that the characteristics of the radio channels are shared among each link of the vertical array. This effect is attributed to the measurement scenario for which scatterers in the elevation plane are scarce in contrast with the azimuth plane. Consequently, it is expected that the distribution of the MPC parameters (i.e., delay and angular spreads) along V is relatively low, as will be demonstrated later in the paper. This effect, critical for the design of RSU arrays, is also confirmed by the similar behavior of the CTF correlation for the same reference antenna in Figure 7b. Figure 8 presents the averaged correlation of $T_{s}$ and the CTF for all antennas of the array along the H and V axis. The results demonstrate that the behavior of the correlation along H and V is highly consistent with the #46 antenna analysis. $\rho_{V}$ is observed to be relatively smooth for all antennas, whereas a periodic variation in $\rho_{H}$ is found, thus suggesting that the spatial properties of the MPC change at a larger rate compared to the array size along H. These findings are in line with previously reported results such as in [21].

### 3.2. Radio Channel Characteristics

It is proposed here to investigate the radio channel characteristics in terms of coherence time and bandwidth as well as delay and angular spread. The purpose of this analysis is to correlate the stationarity properties of the radio channel with the time-varying spatial properties of the MPC.

#### 3.2.1. Coherence Time and Bandwidth

First, it is checked whether the radio channel is doubly underspread. This concept introduced in [11] to characterize non-WSSUS channels states that the CTF is not only slowly varying (dispersion underspread) but that the channel statistics variation is even slower (correlation-underspread). This property can be described by the following inequality:

(8) $$T_{s}F_{s}\gg T_{c}B_{c}\gg 1.$$

**Figure 9:**
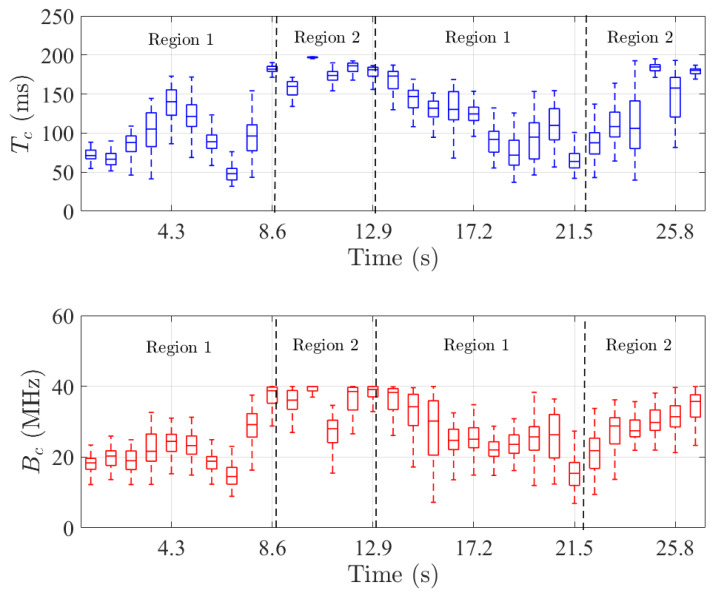
Spatial distribution in boxplot of Tc (blue) and Bc (red) as a function of time.

Thus, the stationarity region is much larger than the coherence region for doubly underspread channels [22]. Figure 9 shows the boxplot spatial distribution of $T_{c}$ and $B_{c}$ as a function of time. First, it is found that $T_{c}$ and $B_{c}$ are always significantly lower than $T_{s}$ and $F_{s}$ with median values from 50 to 190 ms and 10 to 40 MHz, respectively. This confirms the doubly underspread property of the measured scenario. Nonetheless, the data show that the statistics vary as a function of time due to the nature of the investigated scenario. Moreover, $T_{c}$ and $B_{c}$ are found to be highly correlated with a Pearson correlation coefficient of 0.89. To provide a deeper understanding of these variations, the following paragraph explores in detail the large scale parameters of the measured radio channel.

#### 3.2.2. Large Scale Parameters

**Figure 10:**
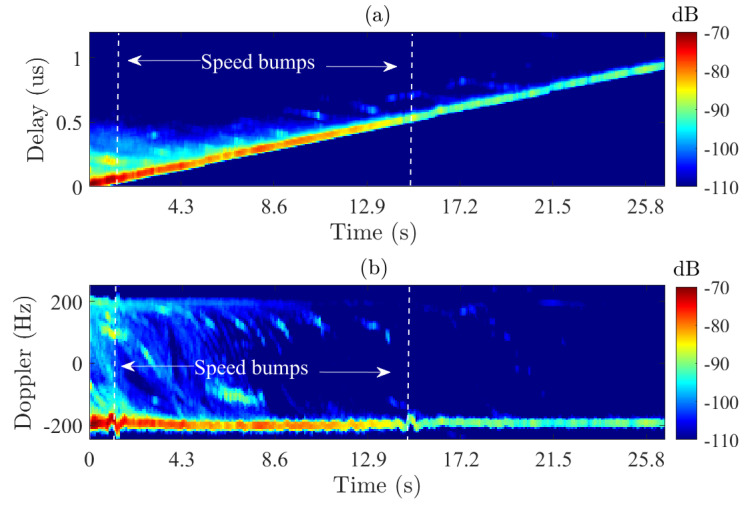
Time-varying *PDP* (**a**) and *DPS* (**b**) averaged across all Tx antennas.

The time-varying *PDP* and *DPS* were then computed from the GLSF within the local stationary region and averaged across all Tx antennas (see Figure 10). The time-varying discrete form of of the *PDP* is computed for each Tx antenna as follows [6,10]:

(9) $$PDP_{l}\left[k_{ts},n\right]=\sum_{p}{\hat{C}}_{l}\left[k_{ts},n,p\right],$$

whereas the time-varying *DPS* is given by [6,10]:

(10) $$DPS_{l}\left[k_{ts},p\right]=\sum_{n}{\hat{C}}_{l}\left[k_{ts},n,p\right].$$

The LOS delay is linearly increasing with a maximum delay of 0.98 μs that is related to the change in the propagation path length of 295 m, and a negative Doppler shift of −200 Hz which is in line with Rx moving away from Tx at a constant speed of 40 km/h. A large set of high-energy MPC can be observed within the first nine seconds with negative and positive Doppler shifts. This is attributed to strong scattering from the front and side buildings during the first half of the driving test, whereas few paths are seen during the second half. In addition, a variation in the Doppler shifts of the LOS is observed at the location of the speed bumps.

**Figure 11:**
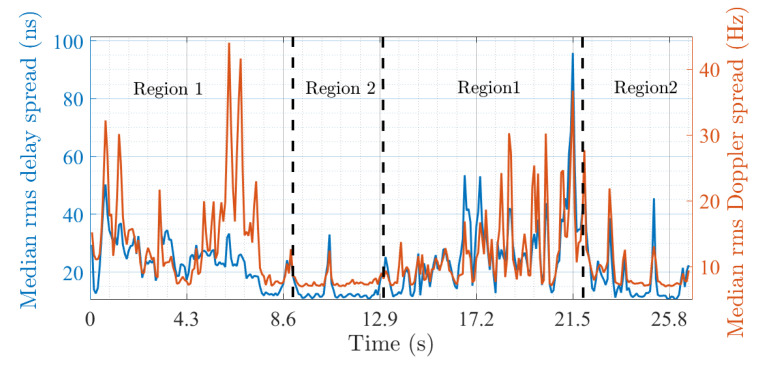
Median rms delay and Doppler spread.

The MPC richness is analyzed from the analysis of the RMS delay and Doppler spread computed after the time-varying *PDP* and *DPS* with a 20 dB threshold [6] (Figure 11). Two regions with specific shadowing characteristics are found that can be physically linked to the geometrical configuration of the investigated scenario (see Figure 2). The first (region 1) corresponds to the first 9 s and between ∼13 and 22 s. The second (region 2) is between ∼9 and 13 s and after 22 s. Region 1 is MPC-rich which can be attributed to buildings surrounding Rx during the first and second half of the driving test. On the other hand, region 2 is MPC-poor and corresponds to the transition in-between due to masking buildings and a lack of strong scatterers. Similarly to $T_{c}$ and $B_{c}$, the results also suggest a high degree of correlation between the delay and Doppler spreads with an average Pearson correlation coefficient of 0.72.

**Figure 12:**
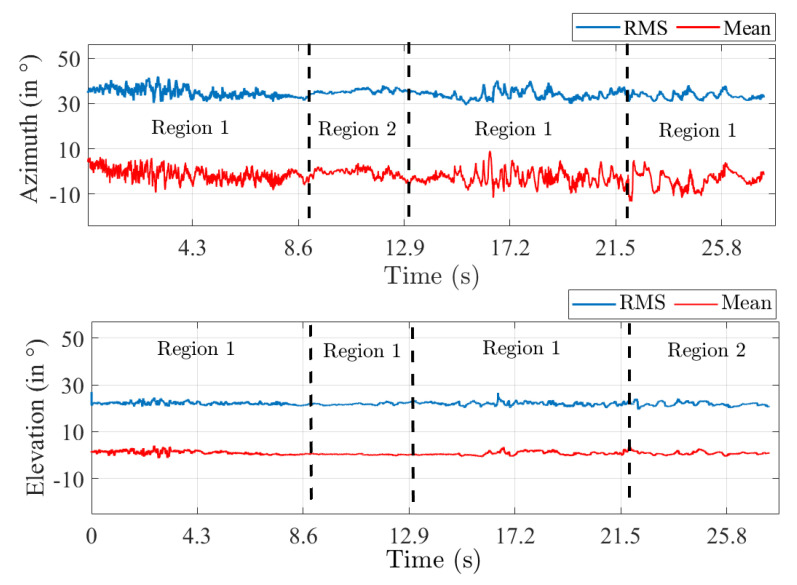
RMS azimuth (**top**) and elevation (**down**) angular spread and mean values.

Finally, the spatial structure of the MPC was investigated by computing the RMS azimuth and elevation angular spreads and associated mean values from the *PDP* using a classical beamforming approach. The results shown in Figure 12 highlight the fact that the MPC geometrical structure does not vary along the driving test. The RMS angular spreads and mean values are found to be nearly constant with time. An RMS of ∼35${}^{\circ }$ and ∼22${}^{\circ }$ in azimuth and elevation was computed on average, respectively. A mean value of ∼0${}^{\circ }$ for both azimuth and elevation is obtained corresponding well to the LOS between Tx and Rx. Nonetheless, the spread values in azimuth exhibit a larger deviation in region 1 compared to region 2 again due to a richer scattering environment. Finally, the spatial correlation along V that was reported earlier can be corroborated by the fact that the spread values are lower in elevation than in azimuth. It can be safely concluded that horizontal arrays are more favorable for this scenario since the MPC richness appears naturally stronger in the horizontal plane.

## 4. Conclusions

The non-WSSUS and large scale characteristics of the vehicle-to-infrastructure V2I link are studied in a suburban environment at the 5.89 GHz vehicular band from measured time-varying single-user mMIMO radio channels. From estimates of the GLSF, the spatial distribution of the stationarity time was evaluated using the CCF and empirical collinearity methods, whereas the root mean square delay/angular spread and coherence time/bandwidth values were computed from the projected *PDP* and *DPS*. The results highlight the high degree of correlation between the spatial distribution of the stationarity time and the scattering properties along the measurement route for which a strong LOS exists. The statistical spatial behavior of the stationarity time is provided along the measurement route across the 64 Tx antennas of the massive MIMO system with an estimated 568 ms median value. It is shown that this spatial distribution is strongly correlated along the array vertical dimension in line with the use of mMIMO linear arrays in previous reports. In addition, within the immediate stationary region, the doubly underspread property of the measured radio propagation channel was confirmed. Finally, for this scenario, a high degree of correlation between the stationarity time characteristics with the scattering properties along the measurement route is highlighted. Future research includes a deeper investigation of the stationarity region and scattering properties for different propagation scenarios at higher vehicle speeds to improve our comprehension of the non-WSSUS mMIMO vehicular channels and develop accurate models.

## Figures and Tables

**Table 1:** MaMIMOSA parameters’ configuration.

Parameter	Configuration
Carrier frequency	5.89 GHz
Measurement bandwidth	80 MHz
Frequency points/Delay resolution	818/12.5 ns
Transmit power	0 dBm
Number of Tx/Rx antenna	64/1
Frame duration	5.504 s (2752 blocks)
Block duration (8 OFDM symbols)	975.36 µs
Interframe/Interblock time	50 ms/1 ms

## Data Availability

Not applicable.
